# An Operational Definition of a Statistically Meaningful Trend

**DOI:** 10.1371/journal.pone.0019241

**Published:** 2011-04-28

**Authors:** Andreas C. Bryhn, Peter H. Dimberg

**Affiliations:** Department of Earth Sciences, Uppsala University, Uppsala, Sweden; University of East Piedmont, Italy

## Abstract

Linear trend analysis of time series is standard procedure in many scientific disciplines. If the number of data is large, a trend may be statistically significant even if data are scattered far from the trend line. This study introduces and tests a quality criterion for time trends referred to as statistical meaningfulness, which is a stricter quality criterion for trends than high statistical significance. The time series is divided into intervals and interval mean values are calculated. Thereafter, r^2^ and p values are calculated from regressions concerning time and interval mean values. If r^2^≥0.65 at p≤0.05 in any of these regressions, then the trend is regarded as statistically meaningful. Out of ten investigated time series from different scientific disciplines, five displayed statistically meaningful trends. A Microsoft Excel application (add-in) was developed which can perform statistical meaningfulness tests and which may increase the operationality of the test. The presented method for distinguishing statistically meaningful trends should be reasonably uncomplicated for researchers with basic statistics skills and may thus be useful for determining which trends are worth analysing further, for instance with respect to causal factors. The method can also be used for determining which segments of a time trend may be particularly worthwhile to focus on.

## Introduction

Searching for meaningful time series trends is an important and common task in scientific work and the statistical significance of a linear trend fit to the time series is often used for classifying the usefulness of a trend [1], [2]. Yet, the statistical significance of a linear trend depends on the number of data analysed. If a large number of data is available, even a weak trend with many data points scattered far away from the trend line and thus with a correlation coefficient (r^2^ value) near zero can be highly significant [3]. Due to the inherent uncertainty in many types of empirical data, it may be practically impossible to conclude that a long and detailed empirical time series is stationary in the sense that there is no significant trend at any reasonable confidence level. There may therefore be a need for a stricter statistical quality indicator for time trends than high statistical significance. The indicator presented in this study will henceforth be referred to as *statistical meaningfulness* and has a dual quality point grading scale: *statistically meaningful*, and *not statistically meaningful*.

There are additional practical reasons why the statistical significance may be an insufficient quality indicator for assessing the usefulness of changes in a time trend. If one scientific phenomenon is represented and measured by several variables, the trends of these variables may have very low p values and may be contradictory if a large enough number of data is used. This problem may be illustrated using data associated with marine eutrophication. The trophic state of a marine (or any other) water body is defined as the level of primary production near the water surface. Primary producers are organisms which use photosynthesis to transform carbon dioxide and water into tissue and other molecules which other organisms can metabolise. In marine waters, primary producers mainly consist of various phytoplankton species. The trophic state is commonly measured by several complementary variables such as chlorophyll-a, nitrogen and phosphorus concentrations. Chlorophyll-a is a phytoplankton pigment while nitrogen and phosphorus are nutrients which individually or in combination control long-term phytoplankton productivity in most marine and estuarine waters. In many cross-systems surveys, total nitrogen, total phosphorus and chlorophyll-a have all been positively correlated in bivariate regressions with r^2^ values typically above 0.65 [3].

**Figure 1:**
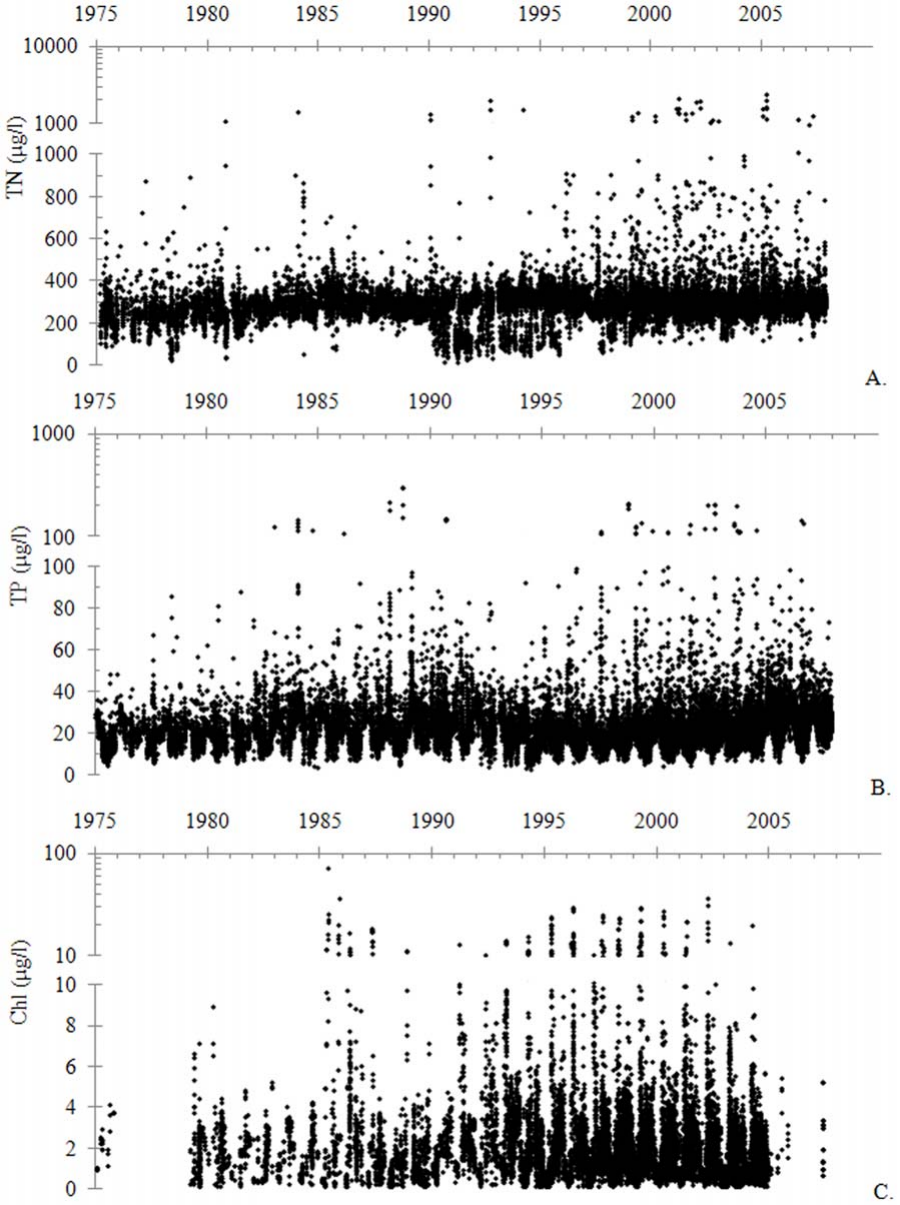
Trophic state indicators in surface waters of the Baltic Sea, 1975–2007. Concentrations in individual samples taken during the whole year. A. Total nitrogen (TN) concentrations; data from [18]. B. Total phosphorus (TP) concentrations; data from [18]. C. Chlorophyll-a (Chl) concentrations; data from [3]. The upper part of the y-axis scales are logarithmic in order to enable representation of the highest values.

Total nitrogen, total phosphorus and chlorophyll-a in surface waters of the Baltic Proper (northern Europe; 54–60°N, 11–23°E), however, showed quite different linear trends during 1975–2007 (fig. 1). The total nitrogen trend increased during this period (r^2^ = 0.003, p<0.001, n = 54,511; fig. 1A) and so did the total phosphorus trend (r^2^ = 0.006, p<0.001, n = 45,609; fig. 1B) although the chlorophyll-a trend decreased (r^2^ = 0.0004, p = 0.010, n = 14,723; fig. 1C). Trends were still sloping at (p<0.05 and in the same directions as described above when years 1975–78 and 2006–07, from which no or few chlorophyll-a measurements are available, were omitted. Because of these contradictory trends it could be difficult to determine whether the trophic state increased, decreased or remained stable in the Baltic Proper during 1975–2007. In other words, it would be useful for scientists, environmental managers and others to be able to determine whether any of the trends in fig. 1 are statistically meaningful, provided that they are statistically significant at some acceptable confidence level. Similar issues could arise in other scientific disciplines.

Admittedly, the perceived or practical meaningfulness of trends may differ greatly between variables even within one specific academic discipline. The aim of this paper is to suggest a definition of a statistically meaningful trend based on previously published statistics concepts and methods and to test this definition on a group of time series from different disciplines. The idea is to establish a method which produces sensible results and which is also simple and straightforward enough to be used by scholars with either basic or advanced statistics skills. Statistical meaningfulness is intended as a complementary quality criterion together with high statistical significance as a basis and support for discussions regarding how a certain time trend should be classified, treated and reported. The Theory section will motivate the definition of a statistically meaningful trend. Details about the analysed time series and about analysis methods will also be provided. Results will be displayed in the subsequent section. The final section of this paper will discuss and conclude the findings and their practical usefulness.

## Materials and Methods

The r^2^ value is commonly known as the coefficient of determination and may be used for measuring the correlation between two variables, x and y. An r^2^ value at or near 0 means low correlation between x and y and a value near 1 indicates high correlation. The r^2^ value is defined in eq. 1 (from [4]):
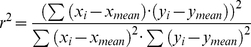
(1)where x_i_ denotes each sequential value in the x data series and and y_i_ represents each value in the y series, while x_mean_ and y_mean_ are the arithmetic mean values of the x and y data series, respectively.

**Figure 2:**
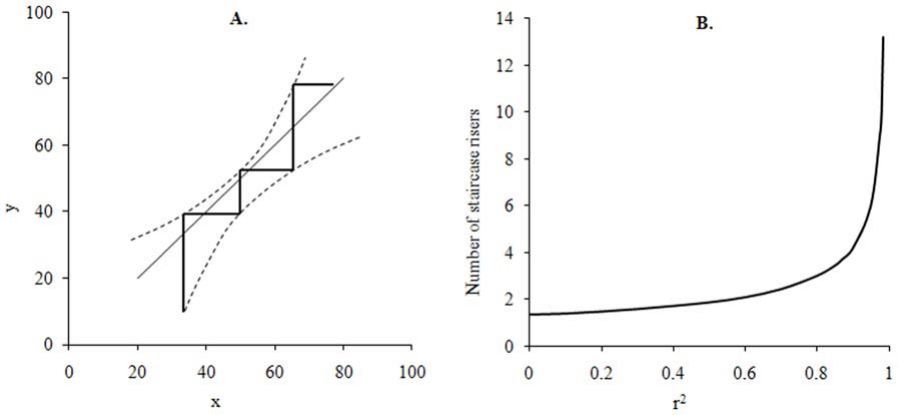
Prairie's staircase, suggesting a non-linear relationship between the correlation coefficient (r^2^) and predictive power. A. The creation of staircase risers from the 95% confidence limits of a correlation. B. The r^2^ value of correlations and the number of obtained staircase risers. From [8].

The p value is used for describing the probability (from 0 to 1) in statistical significance tests in which a null hypothesis is rejected when the p value is low. The 95% confidence level (p≤0.05) has traditionally been used for indicating statistical significance in such tests within a wide variety of academic research fields [3], [5], [6], [7]. The p value of a bivariate regression can be estimated using a statistical table or software relating the p value to the two-tailed Student's t statistic. The degrees of freedom for the p value is then equal to the number of data pairs (n; the number of x data for which corresponding y data are also available) subtracted by 2 (Fisher, 1925). The t value for a bivariate regression is given by n and r^2^ (from [4]):
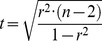
(2)The predictive power of linear regressions has previously been suggested to cross a threshold along the r^2^ value gradient [8]. Fig. 2A shows a regression line and 95% confidence interval for a relationship between two variables, x and y. A solid line is drawn from the lower boundary of the 95% confidence interval from the regression and is drawn upwards until it reaches the upper 95% confidence limit, and is then drawn towards the right until the lower 95% confidence limit is reached again. This procedure is then repeated so that the solid line takes the shape of a staircase (fig. 2A). When Prairie [8] reiterated the exercise in fig. 2A for a large number of correlations, a non-linear relationship was found between r^2^ and the number of staircase risers, and this relationship is depicted in fig. 2B. According to this figure, the typical number of risers is low and similar for r^2^ values from 0 to about 0.65, after which the number rises distinctively. Using the number of staircase risers as a representation of predictive power, Prairie [8] concluded that only linear regressions with r^2^ values higher than 0.65 could yield any useful predictive power. Since its publication, Prairie's predictive power threshold has been used as a statistical benchmark in many scientific books and papers.

Prairie's line of reasoning could also be applied on time series analysis. Using a trend equation and time as a predictor, a variable could be reconstructed with high accuracy if r^2^≥0.65 according to Prairie's staircase method. Moreover, regressions based on mean values over longer time-periods often have higher r^2^ values than those of regressions which include all data pairs [9]. The definition of a statistically meaningful trend will therefore be:

If one or several regressions concerning time and values in a time series, or time and mean values from intervals into which the series has been divided, yields r^2^≥0.65 and p≤0.05, then the time series is statistically meaningful.

This definition uses the well-established 95% confidence level and Prairie's threshold [8] for predictive power. The impact of these limits on statistical meaningfulness test results will, however, be assessed and discussed in the following sections of this study.

**Figure 3:**
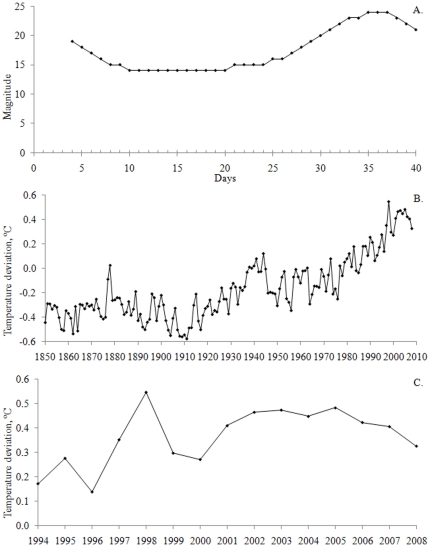
Time series used for testing the statistical meaningfulness definition. A. The midnight magnitude (a unitless brightness measure) of a star during 37 consecutive nights. Data from [10]. B. Global annual temperature deviations 1850–2008 compared to the mean value from the period 1961–1990. Data from [11]. C. Global annual temperature deviations 1994–2008 compared to the mean value from the period 1961–1990. Data from [11].

**Figure 4:**
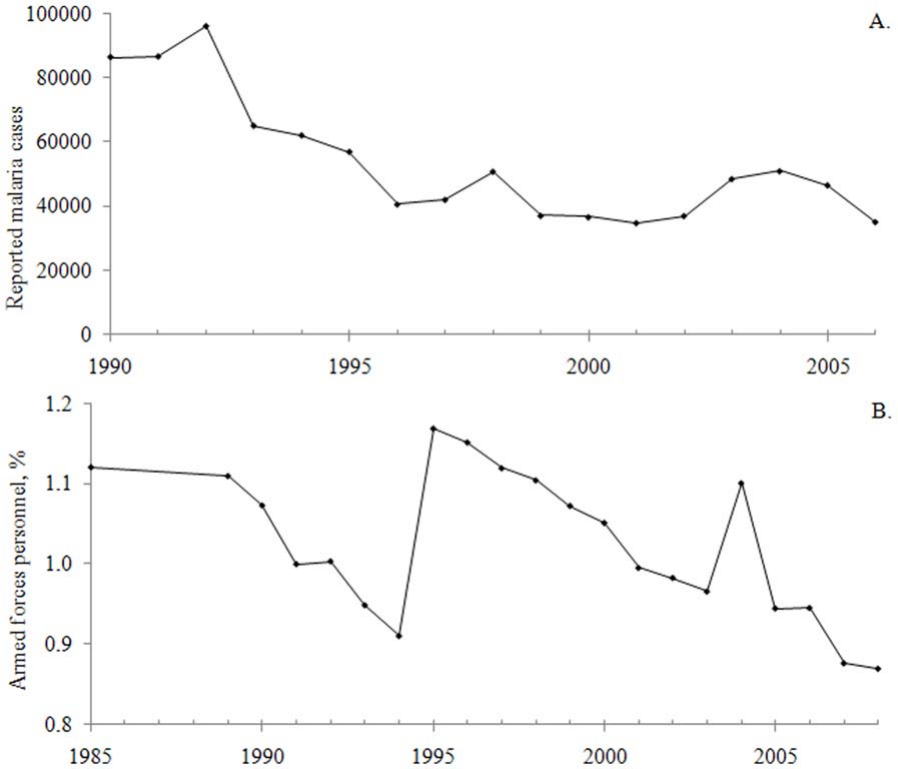
Time series used for testing the statistical meaningfulness definition (continued). A. Reported malaria cases in the Philippines 1990–2006. Data from [12]. B. The 1985–2008 ratio of armed force personnel in the world to the total labour force. Data from [13].

**Figure 5:**
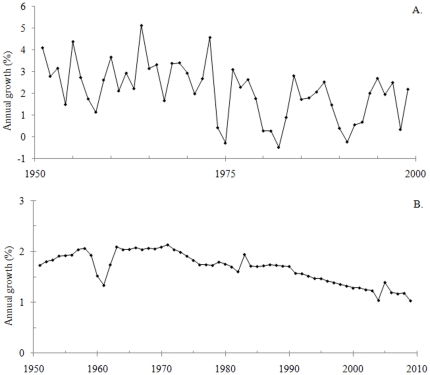
Time series used for testing the statistical meaningfulness definition (continued). A. Annual growth in global aggregate gross domestic product per capita 1951–1999. Data from [14]. B. Annual growth of the global population number 1951–2009. Data from [14].

Ten time series with linear trends at p≤0.05 (table 1) from different academic disciplines (figs. 1 and 3, 4, 5) were used for testing the statistical meaningfulness definition stated in Theory. The three series of chlorophyll, nitrogen and phosphorus concentrations in surface waters of the Baltic Sea are displayed in fig. 1 and were described in the Introduction.

**Table 1:** Test of statistical meaningfulness (p≤0.05 in combination with r^2^≥0.65) using the time series in figs. 1 and 3, 4, 5.

Time series and figure number	Significant trend slope	r^2^ value for full time trend	p value for full time trend	Number of interval divisions indicating statistically meaningful trend	Statistically meaningful trend?
Nitrogen (fig. 1A)	Positive	0.003	<0.001	0	No
Phosphorus (fig. 1B)	Positive	0.006	<0.001	0	No
Chlorophyll (fig. 1C)	Negative	0.0004	0.010	0	No
Star magnitude (fig. 3A)	Positive	0.55	<0.001	0	No
Temperature deviations (fig. 3B)	Positive	0.62	<0.001	**>25**	**Yes**
Temperature deviations (fig. 3C)	Positive	0.30	0.034	0	No
Malaria (fig. 4A)	Negative	0.60	<0.001	**5**	**Yes**
Armed forces personnel (fig. 4B)	Negative	0.27	0.015	**1**	**Yes**
Economic growth (fig. 5A)	Negative	0.22	<0.001	**3**	**Yes**
Population growth (fig. 5B)	Negative	0.63	<0.001	**>25**	**Yes**

The ten time series were divided into 3, 4, 5 and up to 30 intervals. Time was regressed against interval mean values in order to obtain test results. The p and r^2^ statistics for 3 to 19 interval divisions are provided in File S3 (tables S1 and S2). Indications of positive test results have been bolded.

Fig. 3A concerns observations in astronomy and describes the midnight magnitude (i. e., a unitless brightness measure) of a star during 37 consecutive nights (n = 37). Data were taken from [10]. Fig. 3B and 3C display global mean temperature deviation data compared to mean values during 1961–1990, and these climatological data were taken from [11]. These two time series represent temperature deviations 1850–2008 (fig. 3B; n = 159) and 1994–2008 (fig. 3C; n = 15), respectively.

Furthermore, an epidemiology time series on reported malaria cases in the Philippines 1990–2006 was analysed (fig. 4A; n = 17) using data from [12]. The series in fig. 4B (n = 21; from [13]) is related to military science and peace and conflict studies and describes the ratio of armed force personnel in the world to the total labour force 1985–2008.

Finally, economics data from [14] on the percentual growth of the global economy 1951–1999, corrected for the population growth, were used (fig. 5A; n = 49) in addition to demographics data on the percentual numeral increase of the global population 1951–2009 (fig. 5B; n = 59; from [14]).

All time series were divided into intervals using one of the two methods *equal time steps* or *different time steps*. Whenever a time series contained one observation or mean value from each time unit (e. g., day or year), it was divided according to the equal time steps method. An example would be an even time series with seven observations (y_1_–y_7_) at the occasions x_1_ = 1, x_2_ = 2, x_3_ = 3, x_4_ = 4, x_5_ = 5, x_6_ = 6 and x_7_ = 7. If this series should be divided into three intervals, the mean y value from the first interval would be (y_1_+y_2_+1/3 • y_3_)/(2+1/3). The mean y value from the second interval would be (2/3 • y_3_+y_4_+2/3 • y_5_)/(2+1/3). Finally, the mean y value from the third interval would be (1/3 • y_5_+y_6_+y_7_)/(2+1/3). Whenever a time series contained uneven time steps, the series was instead divided into intervals according to the different time steps method. For instance, a time series with seven observations (y_1_–y_7_) at the occasions x_1_ = 1, x_2_ = 3, x_3_ = 4, x_4_ = 5, x_5_ = 6, x_6_ = 8 and x_7_ = 9 could be divided into three time intervals, x = 1–3, 4–6 and 7–9. The mean y values from these intervals would then be (y_1_+y_2_)/2, (y_3_+y_4_+y_5_)/3, and (y_6_+y_7_)/2. Figs. 3, 4A and 5 display time series which were divided into intervals according to the equal time steps method in this study while figs. 1 and 4B show time series with uneven time steps which were divided into intervals using the different time steps method.

To determine which type of interval division is most prone to yielding mean values displaying a statistically meaningful trend according to the definition stated earlier in this section of the paper, Monte Carlo simulations were performed and adapted to producing a Gauss-like distribution shape displaying different occurrences of statistical meaningfulness indications for mean values from different interval divisions. In each simulation run, an x variable was constructed from a series of 1,000 data ranging from 1–1,000 and with an interval of 1, i. e., symbolising a time series with an equal time step of 1 time units between measurements. A y variable was constructed from x and two random variables, Rand1 and Rand2 (both ranging from 0 to 1) according to the following equation:
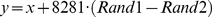
(3)The constant 8281 was calibrated so that the interval division number whose mean values were the most likely ones to indicate a statistically meaningful trend would score positive indications in approximately 50% of the simulation runs. During each simulation run, the time series was divided into intervals of different length according to the equal time step division method. Mean values, p and r^2^ values were calculated and assessed and the statistical meaningfulness was tested.

Monte Carlo simulations were performed using the software Matlab (www.mathworks.com). Trends in time series and correlations were evaluated by means of linear regression using Matlab and Microsoft Excel (office.microsoft.com). A software application for performing the statistical meaningfulness test was programmed and designed in Visual Basic using the Developer Center in Microsoft Excel.

## Results

**Figure 6:**
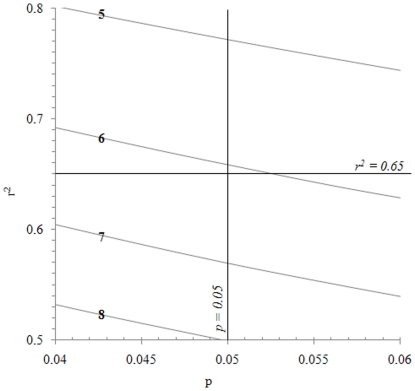
The relationship between the number of data (n; shown on each line in the figure), the probability (p), and the coefficient of determination (r^2^) near p = 0.05 and r^2^ = 0.65. Data were generated by 1,000 Monte Carlo simulation runs.

Equations 1 and 2 give at hand that the number of intervals, the p value and the r^2^ value are interconnected by definition. Results from 1,000 Monte Carlo simulation runs which were intended to visualise the relationship between n, p (near 0.05) and r^2^ (near 0.65) are displayed in fig. 6. If the number of intervals is 7 or more, p≤0.05 may be attained from mean values at r^2^ values below 0.65. Conversely, if the number of intervals is 3–6, regressions with r^2^ values higher than 0.65 may have p values above 0.05.

**Figure 7:**
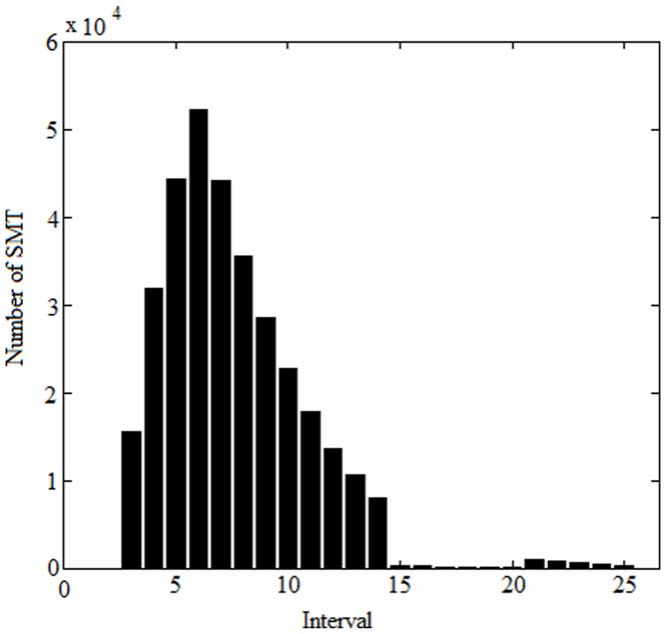
Indication of statistical meaningfulness (SMT; defined as p≤0.05 in combination with r^2^≥0.65) using mean values from different numbers of interval division. Results concerning mean values from 3 to 25 interval divisions are displayed. Series of 1,000 data each were generated by 100,000 Monte Carlo simulation runs and divided into equal intervals.

This breaking point between mean values from 6 and 7 interval divisions initially appeared to play an important role for determining which type of interval division is most prone to producing mean values which indicate a statistically meaningful trend and results from this investigation using 100,000 Monte Carlo simulation runs are displayed in fig. 7. This figure shows which number of interval divisions resulted in mean values indicating statistical meaningfulness regardless of whether or not other numbers of division of the time series produced positive test results. Although the curve in this figure was far from perfectly Gauss-shaped, it clearly revealed that dividing the x and y series into six equal intervals and regressing time against interval means was a comparatively successful way of detecting statistical meaningfulness, as this occurred in 52,154 of the 100,000 simulation runs (fig. 7). Mean values from 5 and 7 interval divisions also gave frequent indications of statistical meaningfulness (in 42,363 and 44,189 runs, respectively). Considerably less reliable indicators were mean values from 8, 4, 9, 10 11, 12, 13 and 14 interval divisions. Divisions into more than 14 intervals produced very few (<1,500) mean values series which indicated statistical meaningfulness.

**Figure 8:**
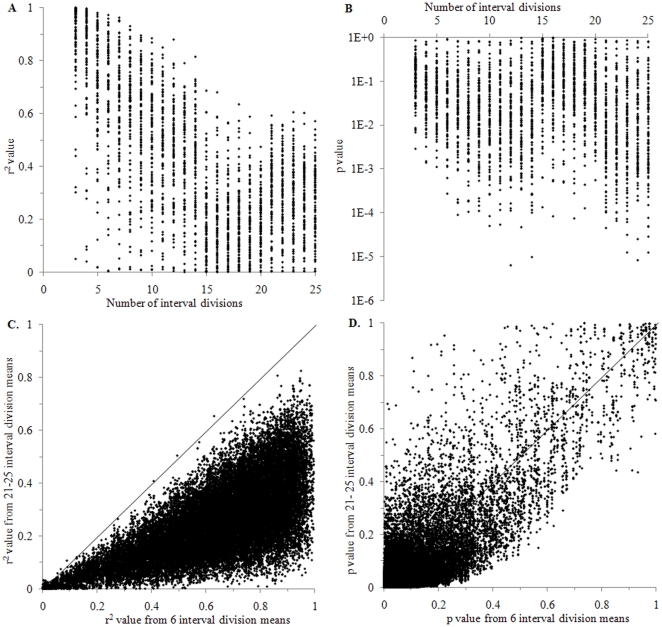
Comparison of r^2^ and p values from interval means between different numbers of intervals into which time series were divided. A. The number of interval divisions in relation to the resulting r^2^ value from time regressed against interval means. Data were generated by 100 Monte Carlo simulation runs; total number of data displayed is 2,300. B. Same as (A), but comparing p values instead of r^2^ values, and with a logarithmic y-axis scale. C. The r^2^ value from six interval division means compared to r^2^ values from 20, 21, 22, 23, 24 and 25 interval division means. The diagonal line marks where x-axis and y-axis values are equal. Data were generated by 6,000 Monte Carlo simulation runs; total number of data displayed is 36,000. D. Same as (C) but comparing p values instead of r^2^ values.

The extent to which the number of intervals and their mean values affect the p and r^2^ values in trends was also studied using the Monte Carlo simulation runs and results are displayed in fig. 8. Fig. 8A shows that the highest r^2^ values were frequently attained using mean values from a small number (3–7) of intervals and that r^2^ values gradually decreased by an increasing number of intervals which the series were divided into. However, exceptions to this general pattern were numerous. The p value displayed an even more obscure pattern in relation to the number of intervals from which mean values and trend regressions were generated (fig. 8B).

In fig. 8C, r^2^ values from time versus interval mean values using six interval divisions are compared to corresponding r^2^ values which were obtained using 20, 21, 22, 23, 24 and 25 interval divisions. In 99.4% of the cases, the interval means from a series divided into six intervals yielded higher r^2^ values than interval means from the larger number of interval divisions on the y-axis of fig. 8C. Whenever this was not the case, r^2^ values were still quite similar (with a difference below 0.04 r^2^ units) and are thus represented just slightly above the diagonal line of fig. 8C. Consequently, mean values from 6-interval divisions of time series could not be used in further tests as a universal indicator of a positive or negative statistical meaningfulness test result. In fig. 8D, p values are compared between mean values of series divided into six intervals and mean values of series divided into 20–25 intervals and these p values appeared to differ greatly but not according to a systematic pattern. Apparently, the number of interval divisions of one and the same time series had a greater impact on the r^2^ value yielded from interval means (figs. 8A and C) than on the p value (figs. 8B and D).

In 19,913 of the 100,000 Monte Carlo simulation runs, means from the 6-interval division indicated that the trend was not statistically meaningful although other division types produced mean values whose trends were statistically meaningful. A careful investigation of results from the simulation runs revealed that it was necessary to take the mean value trend in all interval division types from 3 to 19 interval divisions of each series into account before interval means and p and r^2^ statistics from the remaining division types could be considered redundant.

Altogether, results from the Monte Carlo simulations implied that performing a statistical meaningfulness test could in practice require computer programming skills or very much time spent on manually dividing time series into many different intervals. The authors therefore designed a Microsoft Excel add-in (File S1), i. e., a software application, with the intention to increase the operationality of the statistical meaningfulness test. This add-in (see File S2 for instructions manual and Visual Basics source code) was programmed to automatically perform 3, 4, 5, and up to 30 interval divisions of time series. In addition, the add-in was designed to compute the necessary p and r^2^ values and also determine the statistical meaningfulness of a time series. An interval division and mean values calculation option was added to this application to enable selection between the equal time steps and different time steps methods (see the previous section for an explanation). Using test results from additional interval divisions (>30) in the add-in was also investigated but turned out to impose severe constraints on the possibility to test the statistical meaningfulness of very long time series (containing >40,000 data).

The time series in figs. 1 and 3, 4, 5 were then analysed with respect to p and r^2^ values and to the statistical meaningfulness definition. Interval divisions were made both on-screen in Microsoft Excel and automatically (using Matlab and the add-in), primarily to study and cross-check the statistical meaningfulness in trends but partly also to detect and correct inconsistencies in the add-in. All time series were divided into 3, 4, 5, and up to 100 intervals (or up to the maximum number of intervals for short series) and mean values for each interval were calculated. The main test results from this analysis are displayed in table 1 while the p and r^2^ values which were used for determining the outcome of the test are provided in tables S1 and S2 (File S3). Statistical meaningfulness in trends was detected in five of the time series (table 1; the series displayed in figs. 3B, 4 and 5). In two of these cases (the series in figs. 3B and 5B), interval means from more than 50 interval divisions indicated a statistically meaningful trend while for three of the cases (the series in figs. 4A, 4B and 5A), only five or fewer interval divisions could be used for detecting positive test results (table 1).

Apparently, the limits regarding p and r^2^ which were included in the definition of statistical meaningfulness ultimately determine which ones of the time series presented in this paper may be regarded as statistically meaningful. A sensitivity analysis of these limits is given in table 2. This table was generated using 3, 4, 5 and up to 30 interval divisions of all ten investigated time series. If the r^2^ value limit would be set at 0.85 instead of 0.65, none of the series in figs. 1 or 3, 4, 5would be considered statistically meaningful. Conversely, if the limit would be at 0.55 instead, the series in figs. 1A and 3, 4, 5 would all be classified as statistically meaningful. Likewise, the chosen limit of the p value also affected which time series would pass the test (table 2).

**Table 2:** Sensitivity test of the p and r^2^ limits given in the statistical meaningfulness definition.

Limits	Statistically meaningful time series in figures of this paper
p = 0.05, r^2^ = 0.85	None
p = 0.05, r^2^ = 0.80	3B, 5A, 5B
p = 0.05, r^2^ = 0.75	3B, 5A, 5B
p = 0.05, r^2^ = 0.70	3B, 5A, 5B
p = 0.05, r^2^ = 0.65	3B, 4A, 4B, 5A, 5B
p = 0.05, r^2^ = 0.60	1A, 3A, 3B, 4A, 4B, 5A, 5B
p = 0.05, r^2^ = 0.55	1A, 3A, 3B, 3C, 4A, 4B, 5A, 5B
p = 0.01, r^2^ = 0.65	3B, 4A, 5B
p = 0.1, r^2^ = 0.65	1A, 3A, 3B, 4A, 4B, 5A, 5B

Statistical meaningfulness has been investigated using 3, 4, 5 and up to 30 interval divisions (or up to the maximum number of intervals for short series). The time series which would be regarded statistically meaningful with different limits are listed in the second column. 1A: nitrogen, 3A: star magnitude, 3B: temperature deviations, 3C: temperature deviations, 4A: malaria, 4B: armed force personnel, 5A: economic growth, 5B: population growth.

## Discussion

This study has highlighted and used the well-known fact that high r^2^ values may be attained on behalf of low statistical significance and vice versa [4]. If linear trends are based on all data in a time series, the trend slope may have a very low p value but the spread of data from the trendline may still be high and the r^2^ value may be low (table 1). When time is instead regressed against mean values of different intervals of the time series, r^2^ values tend to increase by a decreasing number of interval divisions (fig. 8, table 1 and table S1 in File S3). The reason why the r^2^ value attains a higher value when a time series is divided into intervals is probably to a large extent due to fewer data with lower variability [9].

When the statistical meaningfulness test was performed on interval mean values from 3, 4, 5 and up to 19 interval divisions of time series generated from 100,000 Monte Carlo simulation runs, results from additional interval divisions became redundant. Consequently, according to our findings, it is necessary and sufficient to take interval means from 3–19 interval divisions of a time series into account in this type of test. The software application (the add-in to Microsoft Excel, see File S1) divides each series into up to 30 intervals, calculates mean values and p and r^2^ statistics and performs the test described in this study. Very few (0.04%) of the 20–25 interval divisions produced r^2^ values from interval means which were higher than corresponding r^2^ values from the six interval division (fig. 8C). Thus, using up to 30 interval divisions should entail sufficient precaution to account for any test results which could have been yielded by a much larger number of Monte Carlo runs than 100,000.

The limits of the r^2^ and p values in the statistical meaningfulness definition fundamentally determine (table 2) which time series should be considered statistical meaningful. The limits advocated in this study (p≤0.05, r^2^≥0.65) have the advantage that they are based on well-established scientific practice. Whether these limits also produce sensible results will be revealed by the manner and extent to which future studies which will use the method presented in this paper.

To the best of our knowledge, there are no previous descriptions in the academic literature of any attempts to establish demarcation criteria for a statistically meaningful time trend which are applicable to many different variables and scientific disciplines and that are stricter than the common requirements regarding high statistical significance. Variable-specific criteria for meaningful trends are, however, plentiful (e. g. [15], [16], [17]). Wu et al. [2] suggested a general method for determining how a time trend should be defined in order to perform appropriate detrending calculations. The present paper could serve as a complement to their method by defining which types of time series are actually meaningful enough to detrend.

To conclude, the present study has presented a method for assessing whether a time series displays a trend which is meaningful enough to analyse further. The method is based on a concept referred to as statistical meaningfulness. To test the rationale for the method, ten time series with linear trends (p<0.05) were described and analysed with respect to statistical meaningfulness and five of the time series were found to be statistically meaningful. A comparison between the temperature deviation time series in figs. 3B and 3C and their characteristics described in table 1 shows that statistical meaningfulness may be present or absent in different spans of a time series; i. e., that some time spans may be more urgent and rewarding to analyse than others. Finally the statistical meaningfulness concept can be used to regard vague and contradictory trends with very low p values such as the ones displayed in fig. 1 and further described in table 1 as not statistically meaningful. The method for determining statistical meaningfulness should be easy enough to use even for researchers with only basic statistics skills, which means that it could become a common and powerful tool in future time series assessment.

## Supporting Information

File S1 **A software application (Microsoft Excel 2007 add-in) which enables a rapid performance of the statistical meaningfulness test.**
(XLAM)

File S2 **Installation manual and source code for File S1.**
(DOC)

File S3 **Two tables containing p and r^2^ values from the statistical meaningfulness test (see** **table 1** **) regarding ten time series described in** **Materials and Methods** **.**
(DOC)
