# Supplementary material for: An Operational Definition of a Statistically Meaningful Trend
Source: PLoS One. 2011 Apr 28;6(4):e19241. doi: 10.1371/journal.pone.0019241 (PMC3084280; doi:10.1371/journal.pone.0019241)
Supplement: File S2 — Installation manual and source code for File S1. (DOC) [file pone.0019241.s002.doc]

**File S2. Supporting information to the article “An operational definition of a statistically meaningful trend” by Andreas C Bryhn and Peter H Dimberg.**

This document concerns the software application (add-in) File_S1.xlam which can be used for performing a statistical meaningfulness test which is described and motivated in the article. This add-in requires Microsoft Excel 2007 or more recent versions of Microsoft Excel. The present document contains a) instructions for installing and using the add-in in Microsoft Excel 2007 (Vista), and b) the source code in Visual Basics and explanations of the source code subroutines, arrays, constants and variables.

**Instructions for installing and operating File_S1.xlam**

1. Download **”File_S1.xlam”** and save it in a suitable computer folder.
2. Open MS Excel 2007 (Vista) and click the Office button **
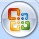
**
3. Select “Excel options”, see figure below.


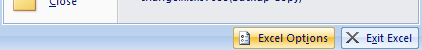


1. Select “Add-ins”
2. Select “OK” next to the option “Handle Excel Add-ins”. Tick the box next to the name **“File S1”** and click “OK”. If “File S1” is not available in the list, click “Browse”, locate the folder and the file. Select the file, double-click and select “OK”.
3. Exit the Office button menu.
4. In Column A in your Excel sheet, you should have, enter or paste the time series which you would like to analyse. In column B, you should have the variable values series which you would like to analyse. Please note a) that the time series must not have any headings, b) that there must be a variable value available for each provided time value; i. e., that there must not be any empty cells in the series, and c) that the time series must be arranged in chronological order.
5. Your toolbar menu should now have the toolbar “Add-ins” available. Select this toolbar.
6. If your time series is a sequence of **equal time steps** (second after second, week after week, year after year or similar), click the icon “E” which should appear like in the figure below.
7. If your time series is a sequence of **different time steps** (for instance, second/day/year number 1, 2, 8, 11, 12, 13, and 18), click the icon “D” which should appear like in the figure below.


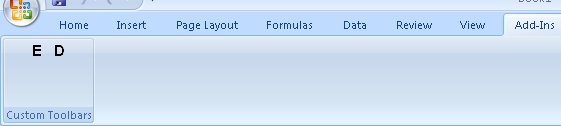


**Source code description and explanation**

File_S1.xlam was designed in the Microsoft Excel Developer Center using the programming language Visual Basics. A Visual Basics code consists of one or several subroutines (“subs”). Constants, strings and variables are often introduced and typologically defined (as, e. g., integers, doubles) using the “Dim” command.

Subroutines in File_S1.xlam

*Sub RemoveBar()*. Removes the command bar for File_S1.xlam if it is already there.

*Sub Auto_Open().* Adds a command bar for File_S1.xlam with two optional icons, E (equal time steps) and D (different time steps).

*Sub Switch1()*. This subroutine is activated when the icon E is clicked to enable the subroutine

*Sub Switch2()*.This subroutine is activated when the icon E is clicked to enable the subroutine

*Sub Equaltimesteps()*. Contains the source code for the equal time steps division method. This subroutine reads the first two columns of the active excel sheet, divides the time series into intervals, calculates interval mean values, r^2^ and p statistics, and performs a statistical meaningfulness test. Output values are added to the user’s active Excel sheet.

*Sub Differenttimesteps()*. Contains the source code for the different time steps division method. This subroutine reads the first two columns of the active excel sheet, divides the time series into intervals, calculates interval mean values, r^2^ and p statistics, and performs a statistical meaningfulness test. Output values are added to the user’s active Excel sheet.

Arrays, constants and variables in File_S1.xlam

*act*. Value: 1 when the subroutine should read a value from the Excel spreadsheet, otherwise value: 0.

*fraction*. In subroutine *Equaltimesteps()*, the remaining fraction of a *segment* whose mean value should be given data in the ongoing calculation step.

*interval*. The number of intervals (from 3 to 30) which the time series should be divided into in the ongoing calculation step.

*intervalmean*. Its final value contains the mean value from all *segment*s of one *interval*.

*intervalmeans*. Array with mean values of each *segment* in an *interval*.

*meaningfulness*. Attains value 1 if the *interval* indicates statistical meaningfulness, otherwise value 0 is attained.

*n.* The time step used in some of the for-loops.

*newsegment.* Value: 1 if calculations for one segment is completed and should start for the subsequent one. Otherwise value: 0.

*numberofdata*. The total length of the analysed time series.

*p*. The p statistic.

*r2*. The r^2^ value.

*segment*. A part of an *interval*; if *interval* = 3 then there will be 3 segments, etc.

*segmentlength*. The length of a *segment*.

*smt*. Value: 1 if *meaningfulness* has ever had a positive value, otherwise value: 0.

*stringfactor*. Strings cannot record and return certain numbers correctly, e. g., very large ones. This variable is related to the largest or smallest number in the series and is used as a factor when certain strings are filled.

*sumofsegmentdata*. The sum of recorded data in one *segment*.

*switch.* Determines whether subroutines *Differenttimesteps()* or *Equaltimesteps()* should be activated.

*t.* The t statistic.

*timemax*. The maximum time value of the series.

*timemin*. The minimum time value of the series.

*usethisinterval*. Value: 1 if the interval can be used for producing mean values, r^2^ values and p values, otherwise *usethisinterval* will have value: 0.

*x*. String which contains the order of each *segment* in an *interval*.

*x2error*. Used for calculating the r^2^ value.

*xmean*. Its final value contains the mean value of the string *x*.

*xyerror*. Used for calculating the r^2^ value.

*y2error*. Used for calculating the r^2^ value.

**Source code for File_S1.xlam**

Sub RemoveBar()

On Error Resume Next

Application.CommandBars("Statistically meaningful trend").Delete

End Sub

Sub Auto_Open()

Dim oToolbar As CommandBar

Dim oButton As CommandBarButton

RemoveBar

Dim MyToolbar As String

Dim switch As Integer

switch = 0

MyToolbar = "Statistically meaningful trend"

On Error Resume Next

Set oToolbar = CommandBars.Add(Name:=MyToolbar, _

Position:=msoBarFloating, Temporary:=True)

If Err.Number <> 0 Then

Exit Sub

End If

On Error GoTo ErrorHandler

Set oButton = oToolbar.Controls.Add(Type:=msoControlButton)

With oButton

.Caption = "Equal time steps"

.OnAction = "Switch1"

.OnAction = "Equaltimesteps"

.Style = msoButtonIcon

.FaceId = 84

End With

Set oButton2 = oToolbar.Controls.Add(Type:=msoControlButton)

With oButton2

.Caption = "Different time steps"

.OnAction = "Switch2"

.OnAction = "Unequaltimesteps"

.Style = msoButtonIcon

.FaceId = 83

End With

oToolbar.Top = 150

oToolbar.Left = 150

oToolbar.Visible = True

Exit Sub

ErrorHandler:

MsgBox Err.Number & vbCrLf & Err.Description

Resume

End Sub

Sub Switch1()

switch = 2

End Sub

Sub Switch2()

switch = -2

End Sub

Sub Equaltimesteps()

If switch < 1 Then

Exit Sub

End If

Cells(1, 4).Value = "Intervals"

Cells(1, 5).Value = "R square"

Cells(1, 6).Value = "p"

Cells(1, 7).Value = "Meaningfulness"

Cells(1, 9).Value = "Statistically meaningful trend?"

Cells(3, 9).Value = "Assuming equal time steps"

Cells(5, 9).Value = "Statistics for entire time series"

Cells(6, 9).Value = "R square"

Cells(6, 10).Value = "p"

Dim timemax As Long

timemax = WorksheetFunction.Max(Columns(1))

Dim timemin As Long

timemin = WorksheetFunction.Min(Columns(1))

Dim numberofdata As Long

numberofdata = WorksheetFunction.Count(Columns(1))

Dim interval As Integer

Dim segment As Integer

Dim x(1 To 100) As String

Dim intervalmeans(1 To 100) As String

stringfactor = WorksheetFunction.Max(Columns(2))

If stringfactor < 0 Then stringfactor = WorksheetFunction.Min(Columns(2))

If stringfactor = 0 Then stringfactor = 1 Else stringfactor = 1 / stringfactor

Dim meaningfulness As Integer

Dim n As Long

Dim act As Integer

Dim segmentlength As Double

Dim fraction As Double

Dim sumofsegmentdata As Double

Dim intervalmean As Double

Dim usethisinterval As Integer

Dim xmean As Double

Dim newsegment As Integer

newsegment = 0

Dim xyerror As Double

Dim x2error As Double

Dim y2error As Double

Dim smt As Integer

Dim t As Double

Dim p As Double

Dim r2 As Double

r2 = WorksheetFunction.RSq(Columns(2), Columns(1))

t = (r2 * (numberofdata - 2) / (1 - r2)) ^ 0.5

p = WorksheetFunction.TDist(t, (numberofdata - 2), 2)

If r2 < 0.65 Then smt = 0 Else smt = 1

If p > 0.05 Then smt = 0

Cells(7, 9).Value = r2

Cells(7, 10).Value = p

For interval = 3 To 30

segmentlength = numberofdata / interval

If segmentlength < 1 Then usethisinterval = 0 Else usethisinterval = 1

xmean = 0

intervalmean = 0

segment = 1

sumofsegmentdata = 0

fraction = 0

For n = 1 To numberofdata

If Cells(n, 1) < timemin Then act = 0 Else act = usethisinterval

If Cells(n, 1) > timemax Then act = 0

If fraction = 0 Then fraction = segmentlength Else act = act

If fraction < 1 Then sumofsegmentdata = sumofsegmentdata + fraction * act * Cells(n, 2) Else

sumofsegmentdata = sumofsegmentdata + act * Cells(n, 2)

If fraction > 1 Then newsegment = 0 Else newsegment = 1

If n = numberofdata Then newsegment = 1

If newsegment = 0 Then

act = act

ElseIf usethisinterval = 0 Then

x(segment) = 0

Else: x(segment) = segment

End If

If newsegment = 0 Then

act = act

ElseIf usethisinterval = 0 Then

intervalmeans(segment) = 0

Else: intervalmeans(segment) = stringfactor * sumofsegmentdata / segmentlength

End If

If usethisinterval * newsegment = 1 Then intervalmean = intervalmean + stringfactor *

sumofsegmentdata / segmentlength / interval Else act = act

If usethisinterval * newsegment = 1 Then xmean = xmean + segment / interval Else act = act

If segment = interval Then

act = act

ElseIf usethisinterval * newsegment = 1 Then

segment = segment + 1

Else: act = act

End If

If usethisinterval * newsegment = 1 Then sumofsegmentdata = 0 Else act = act

If act = 1 Then fraction = fraction - 1

If fraction < 0 Then sumofsegmentdata = sumofsegmentdata - fraction * act * Cells(n, 2)

If fraction < 0 Then fraction = segmentlength + fraction

newsegment = 0

Next

xyerror = 0

x2error = 0

y2error = 0

For n = 1 To interval

If usethisinterval = 0 Then xyerror = 0 Else xyerror = xyerror + (x(n) - xmean) * (intervalmeans(n) –

intervalmean)

If usethisinterval = 0 Then x2error = 0 Else x2error = x2error + (x(n) - xmean) ^ 2

If usethisinterval = 0 Then y2error = 0 Else y2error = y2error + (intervalmeans(n) - intervalmean) ^ 2

Next

If x2error * y2error = 0 Then

r2 = 0

ElseIf xyerror ^ 2 / (x2error * y2error) < 0.000000001 Then

r2 = 0

Else: r2 = xyerror ^ 2 / (x2error * y2error)

End If

If r2 = 1 Then t = t Else t = usethisinterval * (r2 * (interval - 2) / (1 - r2)) ^ 0.5

If r2 = 1 Then

p = 0

ElseIf r2 = 0 Then

p = 1

Else: p = WorksheetFunction.TDist(t, (interval - 2), 2)

End If

If r2 < 0.65 Then meaningfulness = 0 Else meaningfulness = 1

If p > 0.05 Then meaningfulness = 0

Cells(interval - 1, 4).Value = interval

If usethisinterval = 0 Then Cells(interval - 1, 5).Value = "N.A." Else Cells(interval - 1, 5).Value = r2

If usethisinterval = 1 Then Cells(interval - 1, 5).NumberFormat = "General"

If usethisinterval = 0 Then Cells(interval - 1, 6).Value = "N.A." Else Cells(interval - 1, 6).Value = p

If usethisinterval = 1 Then Cells(interval - 1, 6).NumberFormat = "General"

If usethisinterval = 0 Then

Cells(interval - 1, 7).Value = "N.A."

ElseIf meaningfulness = 1 Then

Cells(interval - 1, 7).Value = "Yes"

Else: Cells(interval - 1, 7).Value = "No"

End If

If meaningfulness * usethisinterval = 1 Then smt = 1 Else smt = smt

Erase x

Erase intervalmeans

Next

If smt > 0 Then

Cells(2, 9).Value = "Yes"

Else: Cells(2, 9).Value = "No"

End If

Cells(2, 9).Font.Bold = True

End Sub

Sub Unequaltimesteps()

If switch > -1 Then

Exit Sub

End If

Cells(1, 4).Value = "Intervals"

Cells(1, 5).Value = "R square"

Cells(1, 6).Value = "p"

Cells(1, 7).Value = "Meaningfulness"

Cells(1, 9).Value = "Statistically meaningful trend?"

Cells(3, 9).Value = "Assuming equal time steps"

Cells(5, 9).Value = "Statistics for entire time series"

Cells(6, 9).Value = "R square"

Cells(6, 10).Value = "p"

Dim timemax As Long

timemax = WorksheetFunction.Max(Columns(1))

Dim timemin As Long

timemin = WorksheetFunction.Min(Columns(1))

Dim numberofdata As Long

numberofdata = WorksheetFunction.Count(Columns(1))

stringfactor = WorksheetFunction.Max(Columns(2))

If stringfactor < 0 Then stringfactor = WorksheetFunction.Min(Columns(2))

If stringfactor = 0 Then stringfactor = 1 Else stringfactor = 1 / stringfactor

Dim interval As Integer

Dim segment As Integer

Dim x(1 To 101) As String

Dim intervalmeans(1 To 101) As String

Dim meaningfulness As Integer

Dim n As Long

Dim act As Integer

Dim segmentlength As Double

Dim sumofsegmentdata As Double

Dim numberofsegmentdata As Long

Dim intervalmean As Double

Dim usethisinterval As Integer

Dim xmean As Double

Dim newsegment As Integer

newsegment = 0

Dim xyerror As Double

Dim x2error As Double

Dim y2error As Double

Dim t As Double

Dim p As Double

Dim smt As Integer

Dim r2 As Double

r2 = WorksheetFunction.RSq(Columns(2), Columns(1))

t = (r2 * (numberofdata - 2) / (1 - r2)) ^ 0.5

p = WorksheetFunction.TDist(t, (numberofdata - 2), 2)

If r2 < 0.65 Then smt = 0 Else smt = 1

If p > 0.05 Then smt = 0

Cells(7, 9).Value = r2

Cells(7, 10).Value = p

For interval = 3 To 30

segmentlength = (timemax - (timemin - 1)) / interval

If interval > numberofdata Then usethisinterval = 0 Else usethisinterval = 1

xmean = 0

intervalmean = 0

segment = 1

sumofsegmentdata = 0

numberofsegmentdata = 0

For n = 1 To numberofdata

If Cells(n, 1) < timemin Then act = 0 Else act = 1

If Cells(n, 1) > timemax Then act = 0 Else act = act

If Cells(n, 1) > timemin - 1 + segmentlength * segment Then newsegment = 1

If Cells(n, 1) > timemin - 1 + segmentlength * (segment + 1) Then usethisinterval = 0

If newsegment - numberofsegmentdata = 1 Then usethisinterval = 0

If newsegment = 0 Then sumofsegmentdata = sumofsegmentdata + Cells(n, 2) * act

If newsegment = 0 Then numberofsegmentdata = numberofsegmentdata + act

If n = numberofdata Then newsegment = 1

If newsegment + usethisinterval = 2 Then intervalmeans(segment) = stringfactor * sumofsegmentdata

/ numberofsegmentdata

If newsegment + usethisinterval = 2 Then intervalmean = intervalmean + stringfactor *

sumofsegmentdata / numberofsegmentdata / interval

If newsegment + usethisinterval = 2 Then xmean = xmean + segment / interval

If newsegment + usethisinterval = 2 Then x(segment) = segment

If n = numberofdata Then newsegment = 0

If newsegment + usethisinterval = 2 Then segment = segment + 1

If newsegment + usethisinterval = 2 Then sumofsegmentdata = Cells(n, 2) * act

If newsegment + usethisinterval = 2 Then numberofsegmentdata = act

newsegment = 0

Next

xyerror = 0

x2error = 0

y2error = 0

For n = 1 To interval

If usethisinterval = 0 Then xyerror = 0 Else xyerror = xyerror + (x(n) - xmean) * (intervalmeans(n) –

intervalmean)

If usethisinterval = 0 Then x2error = 0 Else x2error = x2error + (x(n) - xmean) ^ 2

If usethisinterval = 0 Then y2error = 0 Else y2error = y2error + (intervalmeans(n) - intervalmean) ^ 2

Next

If x2error * y2error = 0 Then

r2 = 0

ElseIf xyerror ^ 2 / (x2error * y2error) < 0.000000001 Then

r2 = 0

Else: r2 = xyerror ^ 2 / (x2error * y2error)

End If

If r2 = 1 Then t = t Else t = usethisinterval * (r2 * (interval - 2) / (1 - r2)) ^ 0.5

If r2 = 1 Then

p = 0

ElseIf r2 = 0 Then

p = 1

Else: p = WorksheetFunction.TDist(t, (interval - 2), 2)

End If

If r2 < 0.65 Then meaningfulness = 0 Else meaningfulness = 1

If p > 0.05 Then meaningfulness = 0

Cells(interval - 1, 4).Value = interval

If usethisinterval = 0 Then Cells(interval - 1, 5).Value = "N.A." Else Cells(interval - 1, 5).Value = r2

If usethisinterval = 1 Then Cells(interval - 1, 5).NumberFormat = "General"

If usethisinterval = 0 Then Cells(interval - 1, 6).Value = "N.A." Else Cells(interval - 1, 6).Value = p

If usethisinterval = 1 Then Cells(interval - 1, 6).NumberFormat = "General"

If usethisinterval = 0 Then

Cells(interval - 1, 7).Value = "N.A."

ElseIf meaningfulness = 1 Then

Cells(interval - 1, 7).Value = "Yes"

Else: Cells(interval - 1, 7).Value = "No"

End If

If meaningfulness * usethisinterval = 1 Then smt = 1 Else smt = smt

Erase x

Erase intervalmeans

Next

If smt > 0 Then

Cells(2, 9).Value = "Yes"

Else: Cells(2, 9).Value = "No"

End If

Cells(2, 9).Font.Bold = True

End Sub
