# Supplementary material for: An Operational Definition of a Statistically Meaningful Trend
Source: PLoS One. 2011 Apr 28;6(4):e19241. doi: 10.1371/journal.pone.0019241 (PMC3084280; doi:10.1371/journal.pone.0019241)
Supplement: File S3 — Two tables containing p and r2 values from the statistical meaningfulness test (see table 1 ) regarding ten time series described in Materials and Methods . (DOC) [file pone.0019241.s003.doc]

**File S3. Appendix**

**Table S1. r^2^ values from the statistical meaningfulness procedure test in table 1. The time series used are displayed in figs 1 and 3-5. The ten time series were divided into intervals and time was regressed against interval mean values in order to obtain test results. Indications of positive test results (p ≤ 0.05 in combination with r^2^ ≥ 0.65) have been bolded. N. A. = not applicable (no data available for calculating means from one or several intervals).**

| **Number of interval divisions** | **Nitrogen (fig. 1A)** | **Phosphorus (fig. 1B)** | **Chlorophyll (fig. 1C)** | **Star magnitude (fig. 3A)** | **Temperature deviations (fig. 3B)** | **Temperature deviations (fig. 3C)** | **Malaria (fig. 4A)** | **Armed forces personnel (fig. 4B)** | **Economic growth (fig. 5A)** | **Population growth (fig. 5B)** |
| --- | --- | --- | --- | --- | --- | --- | --- | --- | --- | --- |
| 3 | 0.91 | 0.77 | 0.39 | 0.73 | 0.89 | 0.94 | 0.74 | 0.72 | 0.91 | 0.88 |
| 4 | 0.67 | 0.15 | 0.32 | 0.69 | 0.82 | 0.65 | 0.67 | 0.77 | 0.80 | 0.77 |
| 5 | 0.50 | 0.21 | 0.010 | 0.66 | **0.80** | 0.49 | 0.68 | 0.70 | **0.84** | 0.72 |
| 6 | 0.65 | 0.16 | <0.001 | 0.63 | **0.79** | 0.46 | **0.68** | **0.67** | **0.67** | **0.82** |
| 7 | 0.45 | 0.17 | 0.025 | 0.61 | **0.79** | 0.59 | **0.69** | 0.39 | **0.68** | **0.80** |
| 8 | 0.59 | 0.16 | 0.064 | 0.60 | **0.76** | 0.44 | **0.68** | 0.55 | 0.62 | **0.77** |
| 9 | 0.46 | 0.14 | 0.092 | 0.59 | **0.76** | 0.37 | **0.66** | 0.36 | 0.50 | **0.75** |
| 10 | 0.50 | 0.15 | 0.008 | 0.58 | **0.74** | 0.42 | **0.65** | N.A. | 0.58 | **0.72** |
| 11 | 0.45 | 0.17 | 0.024 | 0.58 | **0.75** | 0.42 | 0.63 | N.A. | 0.61 | **0.74** |
| 12 | 0.53 | 0.15 | 0.024 | 0.57 | **0.74** | 0.37 | 0.64 | N.A. | 0.50 | **0.75** |
| 13 | 0.38 | 0.14 | <0.001 | 0.57 | **0.74** | 0.40 | 0.64 | N.A. | 0.55 | **0.72** |
| 14 | 0.43 | 0.15 | 0.010 | 0.57 | **0.74** | 0.38 | 0.65 | N.A. | 0.53 | **0.71** |
| 15 | 0.35 | 0.14 | 0.058 | 0.57 | **0.71** | 0.30 | 0.64 | N.A. | 0.40 | **0.69** |
| 16 | 0.43 | 0.14 | N.A. | 0.57 | **0.74** | N.A. | 0.63 | N.A. | 0.48 | **0.71** |
| 17 | 0.37 | 0.12 | N.A. | 0.56 | **0.74** | N.A. | 0.60 | N.A. | 0.39 | **0.73** |
| 18 | 0.43 | 0.13 | N.A. | 0.56 | **0.72** | N.A. | N.A. | N.A. | 0.43 | **0.72** |
| 19 | 0.38 | 0.12 | N.A. | 0.56 | **0.73** | N.A. | N.A. | N.A. | 0.38 | **0.69** |

Table S2. p statistics from the statistical meaningfulness procedure test in table 1. The time series used are displayed in figs 1 and 3-5. The ten time series were divided into intervals and time was regressed against interval mean values in order to obtain test results. Indications of positive test results (p ≤ 0.05 in combination with r^2^ ≥ 0.65) have been bolded. N. A. = not applicable (no data available for calculating means from one or several intervals).

| **Number of interval divisions** | **Nitrogen (fig. 1A)** | **Phosphorus (fig. 1B)** | **Chlorophyll (fig. 1C)** | **Star magnitude (fig. 3A)** | **Temperature deviations (fig. 3B)** | **Temperature deviations (fig. 3C)** | **Malaria (fig. 4A)** | **Armed forces personnel (fig. 4B)** | **Economic growth (fig. 5A)** | **Population growth (fig. 5B)** |
| --- | --- | --- | --- | --- | --- | --- | --- | --- | --- | --- |
| 3 | 0.19 | 0.32 | 0.57 | 0.35 | 0.22 | 0.16 | 0.34 | 0.36 | 0.19 | 0.22 |
| 4 | 0.18 | 0.62 | 0.44 | 0.17 | 0.094 | 0.20 | 0.18 | 0.12 | 0.10 | 0.12 |
| 5 | 0.18 | 0.44 | 0.87 | 0.093 | **0.039** | 0.19 | 0.088 | 0.078 | **0.029** | 0.067 |
| 6 | 0.052 | 0.43 | 0.98 | 0.061 | **0.019** | 0.14 | **0.045** | **0.045** | **0.046** | **0.012** |
| 7 | 0.097 | 0.36 | 0.74 | 0.038 | **0.007** | 0.042 | **0.021** | 0.13 | **0.023** | **0.006** |
| 8 | 0.026 | 0.33 | 0.54 | 0.024 | **0.005** | 0.072 | **0.012** | 0.036 | 0.020 | **0.004** |
| 9 | 0.046 | 0.32 | 0.43 | 0.016 | **0.002** | 0.082 | **0.008** | 0.089 | 0.034 | **0.003** |
| 10 | 0.023 | 0.26 | 0.81 | 0.010 | **0.001** | 0.043 | **0.005** | N.A. | 0.010 | **0.002** |
| 11 | 0.024 | 0.20 | 0.65 | 0.007 | **0.001** | 0.032 | 0.003 | N.A. | 0.004 | **0.001** |
| 12 | 0.007 | 0.21 | 0.63 | 0.005 | **<0.001** | 0.037 | 0.002 | N.A. | 0.010 | **<0.001** |
| 13 | 0.024 | 0.21 | 0.96 | 0.003 | **<0.001** | 0.020 | 0.001 | N.A. | 0.004 | **<0.001** |
| 14 | 0.011 | 0.17 | 0.73 | 0.002 | **<0.001** | 0.019 | 0.001 | N.A. | 0.003 | **<0.001** |
| 15 | 0.021 | 0.17 | 0.39 | 0.001 | **<0.001** | 0.034 | <0.001 | N.A. | 0.011 | **<0.001** |
| 16 | 0.006 | 0.15 | N.A. | 0.001 | **<0.001** | N.A. | <0.001 | N.A. | 0.003 | **<0.001** |
| 17 | 0.010 | 0.17 | N.A. | 0.001 | **<0.001** | N.A. | <0.001 | N.A. | 0.007 | **<0.001** |
| 18 | 0.003 | 0.14 | N.A. | <0.001 | **<0.001** | N.A. | N.A. | N.A. | 0.003 | **<0.001** |
| 19 | 0.005 | 0.14 | N.A. | <0.001 | **<0.001** | N.A. | N.A. | N.A. | 0.005 | **<0.001** |
